# Serum biomarker screening and metabolic profiling analysis of nonalcoholic fatty liver disease patients using untargeted metabolomics and machine learning techniques

**DOI:** 10.3389/fmolb.2026.1730023

**Published:** 2026-02-09

**Authors:** Lijuan Dan, Huan Shi, Lina Cao, Xiuyan Li, Lirui Kong, Xiaojie You, Wenping Liu, Yanwei Hao, Dong Wang, Hongfei Song, Jie Mu, Qiao Li

**Affiliations:** 1 Chengdu University of Traditional Chinese Medicine, Chengdu, China; 2 School of Computer and Information Technology, Shanxi University, Taiyuan, China; 3 Clinical Laboratory, West Area of The Third Affiliated Hospital of Chengdu University of Traditional Chinese Medicine, Chengdu, China

**Keywords:** AMPK (AMP-activated protein kinase), differentially expressed metabolites, machine learning, metabolomics, non-alcoholic fatty liver disease

## Abstract

**Background and Objective:**

Non-alcoholic fatty liver disease (NAFLD) represents the most prevalent chronic hepatic metabolic disorder globally. Without timely intervention, it can progress to non-alcoholic steatohepatitis (NASH), liver fibrosis, and even hepatocellular carcinoma. Early detection and diagnosis are critical for disease management. metabolomics, a powerful tool for identifying diagnostic metabolic biomarkers of diseases, is frequently integrated with machine learning (ML) algorithms to improve analytical efficiency. This study aims to compare serum metabolomic profiles between NAFLD patients and healthy controls, identify differential metabolites, and employ machine learning algorithms to discover biomarkers with diagnostic value.

**Methods:**

This study enrolled 26 healthy controls and 165 patients diagnosed with NAFLD via ultrasound, and performed serum untargeted metabolomics analysis. Specifically, metabolomics techniques were used to detect serum metabolites, while orthogonal partial least squares-discriminant analysis (OPLS-DA) was applied to screen for significantly differential metabolites between groups and conduct pathway enrichment analysis. In the ML phase, the dataset was split at an 8:2 ratio: 80% of the data (131 NAFLD cases and 21 healthy controls) was used for model training, and 20% (34 NAFLD cases and five healthy controls) served as an independent test set to validate model performance.

**Results:**

Metabolomic differential analysis identified 942 significantly differential metabolites (656 upregulated and 286 downregulated) between the NAFLD and healthy control groups, which were primarily enriched in caffeine metabolism, cholesterol metabolism, and the FoxO and AMPK signaling pathways. After training and validating machine learning models, serum metabolites maresin 1, canavaninosuccinate, paraxanthine, and 1-methyluric acid demonstrated robust diagnostic performance for NAFLD and can serve as independent predictive biomarkers, with 1-methyluric acid exhibiting the highest diagnostic contribution.

**Conclusion:**

Integration of untargeted metabolomics and machine learning effectively distinguishes NAFLD patients from healthy controls. cholesterol metabolism, caffeine metabolism, and the FoxO and AMPK signaling pathways may participate in NAFLD pathogenesis. ML-validated metabolites 1-methyluric acid, paraxanthine, canavaninosuccinate, and maresin one hold potential as diagnostic biomarkers and therapeutic targets for NAFLD, with 1-methyluric acid exhibiting the highest diagnostic relevance. In summary, serum metabolomics provides stable, accurate biomarkers for NAFLD early warning and diagnosis, and this study offers data and resource support for optimizing its clinical.

## Highlights

- Non-alcoholic fatty liver disease (NAFLD) patients exhibit distinct perturbations in serum metabolite profiles.
- Forkhead box O (FoxO)/AMP-activated protein kinase (AMPK) is the main abnormal metabolic pathway in NAFLD.
- 1-methyluric acid, paraxanthine, canavaninosuccinate, and maresin one are independent predictive markers for NAFLD.

## 1 Introduction

Non-alcoholic fatty liver disease (NAFLD) is a pathological state characterized by hepatic lipid accumulation (Valenti et al., 2009), encompassing a spectrum ranging from simple steatosis, primarily characterized by elevated total cholesterol and triglycerides in the liver, to non-alcoholic steatohepatitis (NASH), characterized by lobular inflammation and hepatocyte damage, and cirrhosis, which may progress to liver failure or hepatocellular carcinoma (Song et al., 2023). Due to sedentary lifestyles, high-calorie diets, and obesity, NAFLD has surpassed viral hepatitis as the leading cause of chronic liver disease globally (Cheemerla and BALAKRISHNAN, 2021). The global prevalence of NAFLD has reached 38%, with a trend toward younger patient demographics, and this prevalence continues to rise in tandem with increasing obesity rates (Younossi et al., 2023; Castera et al., 2019). Epidemiological studies indicate that the prevalence of NAFLD in China increased by 8%–9% from 1999 to 2018, reaching an overall prevalence of 29.1%. Projections indicate that by 2030, the total number of NAFLD patients in China will increase to 314 million, with a prevalence rate of 50.8%, making China the country with the fastest-growing NAFLD prevalence globally (Zhou et al., 2020).

A recent cohort study demonstrated that as NAFLD histological severity increases, the overall mortality risk rises progressively. Notably, even simple hepatic steatosis elevates the risk of death by 71% (Simon et al., 2021). However, public and clinical awareness of its systemic risks remains limited, as patients often seek medical intervention only after symptom onset or, in some cases, after progression to hepatocellular carcinoma (Grech et al., 2015; Golubnitschaja et al., 2012). Liver biopsy remains the gold standard for differentiating between simple steatosis, NASH, and fibrosis stages (Brunt et al., 2021). Conversely, its clinical utility is constrained by inherent risks, sampling variability, high costs, and interobserver variability in histopathological assessment (European AssociationEuropean Association for the Study of Diabetes EASDEuropean Association for the Study of Obesity EASO, 2016). Ultrasonography, a widely adopted non-invasive modality for hepatic steatosis, offers high acceptability and cost-effectiveness. Nevertheless, it exhibits low sensitivity (55%) for mild steatosis at hepatic lipid content <20%, and its diagnostic accuracy is heavily operator-dependent, complicating early-stage detection (Perakakis et al., 2020; Wang et al., 2024). Early diagnosis and intervention are critical to halt NAFLD progression, as lifestyle modifications can reverse early-stage disease and mitigate future healthcare burdens (Chalasani et al., 2018). Current early-detection strategies, however, depend on sparse clinical variables and lack broadly validated biomarkers with high sensitivity and specificity (Zhang et al., 2023). Hence, developing non-invasive diagnostic tools and clinically actionable biomarkers for NAFLD remains an urgent priority.

Metabolomics, an emerging omics technology, enables high-throughput profiling of endogenous small molecules within biological systems. Compared to other omics modalities, metabolomics exhibits closer alignment with phenotypic manifestations, thereby providing mechanistic insights into disease progression and aiding in the discovery and validation of disease-associated biomarkers. Specifically in NAFLD research, metabolomics has been employed to characterize metabolic perturbations involving fatty acids, amino acids, and carbohydrates (Dumas et al., 2014). Metabolic biomarkers are extensively utilized to elucidate disease mechanisms, facilitate diagnosis, and monitor therapeutic responses (Griffiths et al., 2010). As defined by the U.S. Food and Drug Administration, a biomarker is a characteristic that can be objectively measured and evaluated as an indicator of normal biological processes, pathogenic mechanisms, or pharmacological responses to therapeutic interventions (Silver Spring 2016). While metabolomics can quantify hundreds to thousands of metabolites in clinical specimens and discover candidate biomarkers for NAFLD diagnosis and prognosis, challenges persist in metabolite annotation and high-dimensional data interpretation. Machine learning (ML) algorithms, a subset of artificial intelligence, excel at discerning patterns within complex datasets and predicting outcomes. Supervised ML algorithms, including K-Nearest Neighbors (KNN), Random Forest (RF), Support Vector Machines (SVM), Gaussian Naive Bayes (GNB), Logistic Regression (LR), and Decision Trees (DT), can efficiently mine metabolomic datasets to identify discriminative metabolites and develop predictive classifiers, thereby enhancing research efficiency and diagnostic accuracy. In this study, we integrated untargeted metabolomics with ML algorithms to delineate the metabolic signatures of NAFLD and identify potential biomarkers, aiming to provide a foundation for early diagnosis and precision medicine strategies in NAFLD management.

## 2 Methods

### 2.1 Study population

This study enrolled 165 patients with NAFLD and 26 healthy controls. All participants underwent standardized Doppler ultrasound examinations performed by a consistent team of experienced sonographers following a uniform protocol. Blood samples were collected from the Health Examination Centre of Chengdu Pidu District Traditional Chinese Medicine Hospital between December 2024 and January 2025. Participants met the following inclusion criteria: 1) No history of chronic excessive alcohol consumption (ethanol intake: <30 g/day for males, <20 g/day for females); 2) Absence of other etiologies of fatty liver disease (e.g., chronic viral hepatitis, autoimmune hepatitis, drug-induced liver injury, Wilson’s disease, total parenteral nutrition, inflammatory bowel disease, Cushing’s syndrome, celiac disease); 3) Ultrasonographic features consistent with hepatic steatosis (enhanced anterior hepatic echogenicity, posterior acoustic attenuation, and poor visualization of intrahepatic biliary structures). Exclusion criteria were: 1) Pregnancy or lactation; 2) Concurrent cirrhosis or abnormal liver function (defined as alanine aminotransferase (ALT) or aspartate aminotransferase (AST) levels exceeding 3 times the upper limit of normal, or gamma-glutamyl transferase (GGT) levels exceeding 5 times the upper limit of normal); 3) Comorbid severe malignancies (including digestive, immune, and circulatory systems); 4) Recent or current use of systemic corticosteroids, anti-inflammatory agents, hypoglycemic/lipid-lowering medications, or antihypertensive drugs; 5) Incomplete medical records that could not be validated. This study adhered to the ethical guidelines of the Declaration of Helsinki and was approved by the Ethics Review Committee of Chengdu Pidu District Traditional Chinese Medicine Hospital (Ethics Approval No: K-2024-043). All participants provided written informed consent after fully understanding the study objectives and potential risks.

### 2.2 Chemicals, reagents, and equipment

LC-MS grade methanol (MeOH) was purchased from Fisher Scientific (Loughborough, UK). 2-Amino-3-(2-chlorophenyl) propionic acid was obtained from Aladdin (Shanghai, China). Ultrapure water was generated using a Milli-Q system (Millipore, Bedford, USA). A high-speed centrifuge was obtained from Hunan Xiangyi Experimental Equipment Co., Ltd. (Hunan, China). A centrifugal vacuum evaporator was sourced from Eppendorf China Ltd. (Shanghai, China). A vortex mixer was purchased from Haimen Kylin-bell Lab Instruments Co., Ltd. (Haimen, China). Microporous membrane filters (0.22 µm) were obtained from Tianjin Jinteng Experimental Equipment Co., Ltd. (Tianjin, China).

### 2.3 Serum sample collection and metabolite extraction

#### 2.3.1 Serum sample collection

After an overnight fast of at least 12 h, blood samples were collected from all participants via venous puncture in the early morning for metabolomics analysis. Peripheral blood samples were centrifuged at 3,000 *g* for 15 min at 4 °C to separate serum, which was aliquoted into Eppendorf tubes. Samples were then inventoried, stored at −80 °C, and strictly protected from freeze-thaw cycles until metabolomics analysis, to minimize metabolite degradation.

#### 2.3.2 Metabolite extraction

Serum samples stored at −80 °C were subjected to the following pretreatment steps: Experimental samples were first thawed at 4 °C and vortexed thoroughly. Subsequently, 60 µL of each sample was mixed with 400 µL of extraction solution (acetonitrile: methanol = 1: 4, v/v) and transferred to a 2 mL centrifuge tube. The mixture was centrifuged at 12,000 rpm for 15 min at 4 °C. The supernatant was collected, resuspended in 150 µL of 80% methanol aqueous solution containing 4 ppm 2-chloro-L-phenylalanine, filtered through a 0.22 µm membrane, and the filtrate was transferred to a detection vial for liquid chromatography-mass spectrometry (LC-MS) analysis.

### 2.4 LC-MS analysis and data preprocessing

#### 2.4.1 LC-MS analysis

##### 2.4.1.1 Chromatographic conditions

The Thermo Vanquish ultra-high-performance liquid chromatography (UHPLC) system (Thermo Fisher Scientific, USA) was employed for separation using a 2.1 mm × 100.0 mm ACQUITY UPLC HSS T3 column (Waters, Milford, MA, USA) maintained at 25 °C. Mobile phases comprised 0.1% formic acid in acetonitrile (B2) and 0.1% formic acid in water (A2) for positive ion mode, and acetonitrile (B3) and 5 mM ammonium formate in water (A3) for negative ion mode. The linear gradient elution program was as follows: 0–1 min, 10% B3; 1–5 min, 10 %–98% B3; 5–6.5 min, 98% B3; 6.5–6.6 min, 98 %–10% B3; 6. six to eight min, 10% B3 (Zelena et al., 2009).

##### 2.4.1.2 Mass spectrometry conditions

The Thermo Orbitrap Exploris 120 mass spectrometer (Thermo Fisher Scientific, USA) equipped with an electrospray ionization (ESI) source was used. For untargeted metabolomics analysis, the positive ion mode utilized a spray voltage of 3.50 kV, whereas the negative ion mode employed −2.50 kV. The sheath gas and auxiliary gas were set at 40 and 10 arbitrary units (arb), respectively. The capillary temperature was maintained at 325 °C. The primary full scan resolution was set to 60,000 with an ion scan range of m/z 100–1,000. Higher-energy collisional dissociation was employed for MS^2^ fragmentation with a normalized collision energy of 30%, a secondary resolution of 15,000, and acquisition of the top four most abundant precursor ions. Dynamic exclusion was enabled to eliminate redundant MS/MS acquisitions (Want et al., 2013).

#### 2.4.2 Data preprocessing and metabolite annotation

The untargeted metabolomics raw data (including positive and negative ionization modes) from this study have been deposited in the OMIX database (maintained by the National Genomics Data Center) under accession numbers OMIX014425 and OMIX014426. The final dataset, including peak identifiers, sample annotations, and normalized peak areas, was imported into the R XCMS (v3.12.0) package for multivariate analysis (Rasmussen et al., 2022). Orthogonal partial least squares discriminant analysis (OPLS-DA) was performed to visualize group separation and screen for differential metabolites. Model robustness was validated using 7-fold cross-validation and 200 permutation tests to mitigate overfitting. Model performance was evaluated using R^2^Y and Q^2^ metrics, where values approaching one indicate better goodness of fit and predictive accuracy for the training set. Metabolite identification was performed using public databases (HMDB, MassBank, LipidMaps, mzCloud, KEGG) and our proprietary spectral library. Primary MS matching used accurate parent-ion m/z values with a mass deviation threshold of ≤20 ppm to predict molecular formulas and enable preliminary database matching. For metabolites with MS/MS data, fragment-ion spectra were matched against database entries using a threshold of ≥70% and a retention-time tolerance of ≤0.5 min for validation. This workflow conforms to Level 2 (Presumptive Identification) as defined by the Metabolomics Society, meeting the requirement for database-based spectral matching. Metabolites with variable importance in projection (VIP) scores >1 and *P* < 0.05 were considered significantly altered. Identified differential metabolites were imported into MetaboAnalyst for Kyoto Encyclopedia of Genes and Genomes (KEGG) pathway enrichment analysis to identify significantly perturbed metabolic pathways.

### 2.5 Machine learning analysis

#### 2.5.1 Data preprocessing

For algorithms sensitive to feature scaling, including KNN, SVM, and LR, the StandardScaler from scikit-learn was applied to standardize the preselected core features. This ensured all features were on a comparable scale, mitigating the impact of varying numerical ranges on model training.

#### 2.5.2 Feature selection

RF, a widely adopted ensemble learning algorithm, was employed to assess feature importance and interactions (Ding et al., 2023). Leveraging its robust handling of non-linear relationships and high-dimensional data, the RF classifier was trained to compute Gini impurity-based importance scores for each feature. The top 10 features with the highest scores were selected as core features, and their importance rankings were visually presented using a bar chart.

#### 2.5.3 Model training and validation

Six machine learning algorithms were implemented to develop predictive models based on the selected core features: KNN, RF, SVM, GNB, LR, DT. The dataset (n = 191) was partitioned into an 80% training set and a 20% test set using stratified sampling to maintain class distribution. Model performance was evaluated using six metrics: accuracy, sensitivity, positive predictive value (PPV), negative predictive value (NPV), F1-score, and area under the receiver operating characteristic curve (AUC). All analyses were conducted in PyCharm (v2025.1.3) with Python 3.x, leveraging scikit-learn (v1.3.2) for algorithm implementations. Comparative analysis of these models validated the effectiveness of the selected core features for metabolomics data classification. Additionally, to mitigate overfitting, feature leakage, and validation bias under small-sample conditions, this study employs a multi-strategy validation approach: it evaluates model performance stability through 5-fold cross-validation, analyzes performance dispersion in small samples using box plots of 5-fold cross-validation results, compares the consistency of AUC validation strategies between 5-fold cross-validation and leave-one-out validation, and combines leave-one-out AUC distributions with bootstrap validation to assess the model’s small-sample generalization capabilities.

#### 2.5.4 SHAP analysis

SHAP (SHapley Additive exPlanations), a model-agnostic interpretation method rooted in Shapley values from game theory, quantifies the contribution of each feature to the model’s predictions. It has been validated as an effective tool for dissecting the predictive mechanisms of machine learning models in metabolomics research. To identify features with significant predictive relevance to outcomes, important features were prioritized based on their average absolute SHAP values. The core features selected in each analysis, along with their average absolute SHAP contribution values, were reported to pinpoint the key serum metabolites that distinguish NAFLD from healthy controls.

### 2.6 Statistical analysis

In this study cohort, the Mann-Whitney U test and χ^2^ test were applied to compare differences in population characteristics between the healthy control group and NAFLD group for continuous and categorical variables, respectively. Student’s t-test was used to analyze differences in metabolic profiles between the two groups, with P < 0.05 considered statistically significant.

## 3 Results

### 3.1 Characteristics of the study participants

After rigorous screening based on inclusion and exclusion criteria, a total of 191 participants were enrolled in this study, including 165 in the NAFLD group and 26 in the healthy control group. The NAFLD group comprised138 males and 27 females, with an age range of 22–66 years and a mean age of 40.31 ± 10.15 years; ALT and AST levels in this group were 46.03 ± 30.01 U/L, 29.29 ± 13.32 U/L, respectively. The healthy control group consisted of seven males and 19 females, with an age range of 24–43 years and a mean age of 36.5 ± 7.10 years; its corresponding serum ALT and AST levels were 23.65 ± 19.36 U/L and 21.00 ± 7.61 U/L. Statistical analysis revealed a significant difference in gender distribution, ALT, and AST between the two groups (*P* < 0.05), with the proportion of males in the NAFLD group being significantly higher than that in the healthy control group. In contrast, no statistical difference was observed in age distribution between the two groups (*P* > 0.05).

### 3.2 Identification of differential metabolites between NAFLD patients and healthy controls

#### 3.2.1 OPLS-DA model construction

**FIGURE 1:**
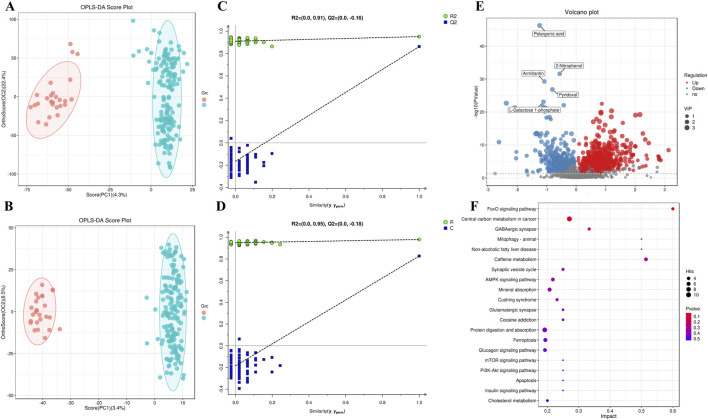
**(A)** OPLS-DA score scatter plot of the NAFLD group vs. healthy controls (positive ion mode); **(B)** OPLS-DA score scatter plot of the NAFLD group vs. healthy controls (negative ion mode); **(C)** 200-permutation test for the OPLS-DA model (positive ion mode); **(D)** 200-permutation test for the OPLS-DA model (negative ion mode); **(E)** Volcano plot comparing the NAFLD group and healthy controls; **(F)** KEGG classification of differential metabolites between the NAFLD group and healthy controls.

OPLS-DA model was applied to compare metabolic profiles between the NAFLD group and healthy controls. Figures 1A,B present the results of supervised OPLS-DA analysis, which visually demonstrate distinct separation between the two groups. Model reliability was validated via permutation tests (Figures 1C,D), confirming that the OPLS-DA models were stable, reliable, and free from overfitting. To evaluate goodness of fit and predictive performance, cumulative R^2^Y and Q^2^ values were calculated for the OPLS-DA models. In positive ion mode, the fitting metrics were R^2^Y = 0.952 (*P* < 0.05) and Q^2^ = 0.863 (*P* < 0.05); in negative ion mode, the metrics were R^2^Y = 0.979 (*P* < 0.05) and Q^2^ = 0.827 (*P* < 0.05). These results indicate low overfitting risk and robust predictive capacity across both ion modes, supporting their suitability for subsequent metabolic biomarker screening.

#### 3.2.2 KEGG enrichment analysis of differentially metabolised substances

A total of 7,462 metabolites were identified across both positive and negative ion modes. Among these, 2,850 metabolites were structurally annotated via MS^2^ spectral matching and confidently assigned chemical names for downstream analysis. Potential differential metabolites were screened using VIP scores >1 and *P* < 0.05, with permutation tests confirming the absence of overfitting in the OPLS-DA models. Volcano plots were generated to visualize differential expression (Figure 1E). Compared to healthy controls, 942 differential metabolites were identified in the NAFLD group, including 656 upregulated and 286 downregulated metabolites. These metabolites primarily comprised lipid and lipid-like molecules, organic heterocyclic compounds, organic acids and derivatives, organic oxygen compounds, organic sulfur compounds, organometallic compounds, organic nitrogen compounds, organic 1,3-dipolar compounds, alkaloids and derivatives, lignans, neolignans and related compounds, nucleosides, nucleotides and analogues, and benzene derivatives. KEGG pathway enrichment analysis of these 942 differential metabolites identified significantly perturbed metabolic pathways, primarily involving cholesterol metabolism, caffeine metabolism, ferroptosis, animal mitophagy, apoptosis, as well as the Forkhead box O (FoxO) signaling pathway, AMP-activated protein kinase (AMPK) signaling pathway, mTOR signaling pathway, and PI3K-Akt signaling pathway (Figure 1F).

### 3.3 ML-driven discovery of metabolic biomarkers for NAFLD

**FIGURE 2:**
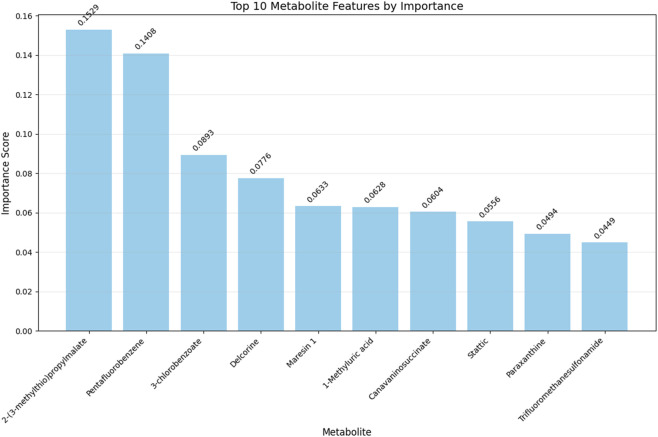
Top ten important metabolites screened by the random forest regression model.

**FIGURE 3:**
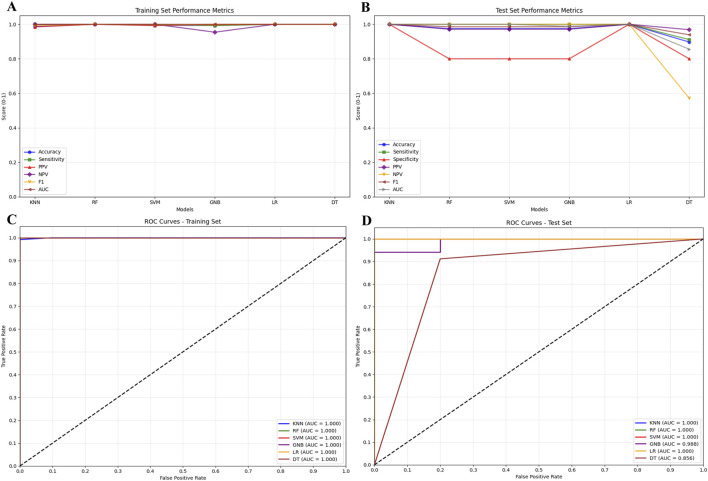
**(A)** Performance metrics of the training set (accuracy, sensitivity, PPV, NPV, F1 score); **(B)** Performance metrics of the test set (accuracy, sensitivity, PPV, NPV, F1 score); **(C)** ROC curve of the training set (model predictive performance validation); **(D)** ROC curve of the test set (model generalizability validation).

To mitigate overfitting risk in machine learning caused by the imbalance between 942 differential metabolites identified via untargeted metabolomics screening and the limited sample size (n = 191), this study employed OPLS-DA for rigorous feature selection. The VIP threshold was elevated from the conventional one to 2.8, ultimately narrowing down to 19 core differential metabolites (Supplementary Table S1). A random forest regression model was then used to train and process the feature data, identifying the top 10 important metabolites (Figure 2). For model performance evaluation, the 191 samples were split into a test set and training set at a 2:8 ratio, and six machine learning models were constructed using KNN, RF, SVM, GNB, LR, and DT algorithms (Figures 3A,B). Among these, the LR model exhibited optimal performance in both sets, with sensitivity, specificity, and AUC all reaching 1 (Figures 3C,D); the remaining five models also showed good predictive performance, confirming that these metabolites can serve as biomarkers for NAFLD identification.

**FIGURE 4:**
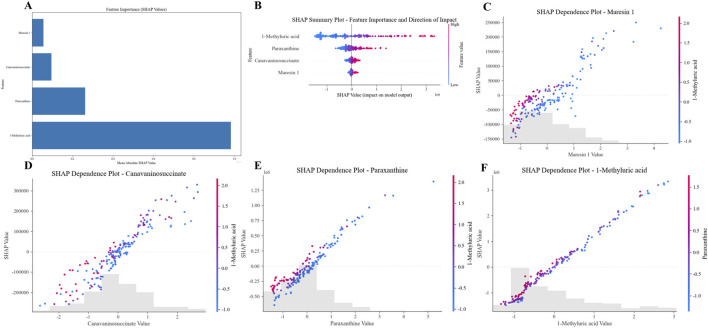
**(A)** Feature importance ranking plot; **(B)** SHAP beeswarm plot of key features; **(C)** SHAP value dependence plot for maresin 1; **(D)** SHAP value dependence plot for canavaninosuccinate; **(E)** SHAP value dependence plot for paraxanthine; **(F)** SHAP value dependence plot for 1-methyluric acid.

For the 10 selected metabolic features, after excluding exogenous interference based on literature evidence, we analyzed their impact on model decision-making using interpretable methods, including feature importance ranking plots (Figure 4A), SHAP summary plots (Figure 4B), and dependency plots (Figures 4C–F). Feature importance ranking revealed that 1-Methyluric acid exhibited the highest contribution and dominated model decisions. The SHAP summary plot further indicated that 1-methyluric acid, paraxanthine, canavaninosuccinate, and maresin one influenced classification via bidirectional regulation. For example, the broad distribution of SHAP values for 1-methyluric acid demonstrated that high and low values affected model outputs in opposing directions. In the single-feature SHAP dependency plot, as the concentration of 1-methyluric acid increased, its SHAP value also increased, contributing more strongly to positive class predictions. Additionally, other metabolites such as paraxanthine modulated this relationship, illustrating how metabolite interactions influenced model classification. Collectively, 1-methyluric acid emerged as the most diagnostically valuable biomarker, with concentration changes influencing disease classification through metabolic networks and exhibiting significant nonlinear synergistic effects with paraxanthine, canavaninosuccinate, and maresin 1.

**FIGURE 5:**
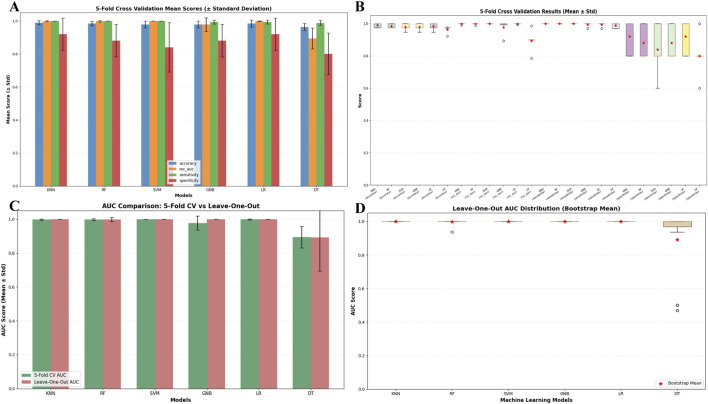
**(A)** Comparison of model performance metrics (Mean ± Standard deviation) via 5-fold Cross-Validation; **(B)** box plot of model performance metric distributions via 5-fold cross-validation; **(C)** comparison of model AUC metrics between 5-fold cross-validation and leave-one-out; **(D)** leave-one-out cross-validation AUC distribution box plot with bootstrap means for machine learning models.

To address risks of overfitting, feature leakage, and validation bias under small-sample conditions, this study employs multi-strategy validation to assess model performance: Five-fold cross-validation was conducted on the accuracy, AUC, sensitivity, and specificity of KNN, RF, SVM, GNB, and LR models; results show that the mean values of all metrics are ≥0.95 with standard deviations ≤0.02 (Figure 5A), demonstrating robust performance stability. Box plots of 5-fold cross-validation results (Figure 5B) reveal that core metrics like accuracy and AUC for KNN, RF, and SVM models cluster within the 0.95–1.0 range without low-scoring outliers, indicating that small samples did not cause performance dispersion. The AUC comparison between 5-fold cross-validation and leave-one-out validation (Figure 5C) indicates that the mean AUC values for the aforementioned core models under both strategies are ≥0.98, with no significant differences in error bars, validating consistent performance across different strategies. The AUC distribution plot for the leave-one-out method (Figure 5D) shows no data points below 0.8 for the core models’ AUC; combined with the mean value close to 1.0 calculated using the bootstrap method, this further confirms that the small sample size did not compromise the models’ stable recognition capability for scarce samples.

**FIGURE 6:**
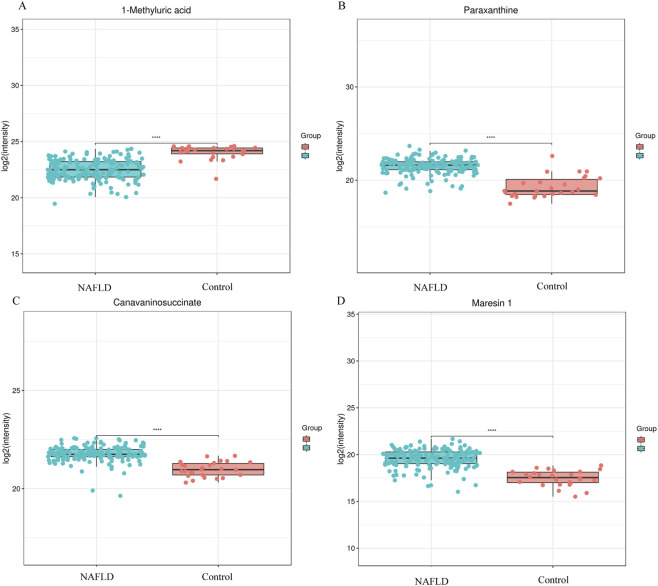
Intergroup box plots of machine learning screening of core metabolites **(A)** 1-methyluric acid; **(B)** paraxanthine; **(C)** canavaninosuccinate; **(D)** maresin one.

To visually characterize the distribution patterns of the core differential metabolites identified through machine learning-based screening in the NAFLD group and the healthy control group, box-and-whisker plot visualization was conducted for four key metabolites, namely, 1-methyluric acid, paraxanthine, canavaninosuccinate, and maresin 1 (Figures 6A–D). The results revealed that the Log_2_ (intensity) of 1-methyluric acid was significantly lower in the NAFLD group (represented by blue dots) compared to the control group (represented by red dots, *P* < 0.0001). For paraxanthine, canavaninosuccinate, and maresin 1, their Log_2_ (intensity) levels were notably higher in the NAFLD group than in the control group (*P* < 0.0001, with scatter points concentrated in the upper half of the boxes). The expression discrepancies of these metabolites between the two groups were consistent with the screening outcomes of the machine learning model, thus validating the biological relevance of the core features. Specifically, the low expression of 1 - methyluric acid and the high expression of the other three metabolites can function as metabolic phenotypic indicators for distinguishing NAFLD from healthy controls.

## 4 Discussion

NAFLD represents the most prevalent chronic metabolic liver disease globally, with its pathogenesis encompassing multiple dimensions including glucose, lipid, and cholesterol metabolism dysregulation, inflammatory responses, and hepatocellular injury (Yan et al., 2022). Hepatitis, cirrhosis, and hepatocellular carcinoma secondary to NAFLD have evolved into critical global public health concerns. Over the past 2 decades, concurrent with China’s rapid economic expansion and profound lifestyle transformations, the disease burden of NAFLD has escalated substantially. Despite extensive investigations into NAFLD, the core molecular mechanisms governing its etiology and pathogenesis remain incompletely elucidated (Ye et al., 2022). Thus, in-depth dissection of NAFLD pathogenesis, identification of relevant biomarkers, and provision of novel theoretical frameworks for its early diagnosis, prevention, and targeted therapeutics have become imperatives. Metabolomics, by profiling intermediates and end products of diverse metabolic pathways, furnishes direct phenotypic signatures and tissue-/time-resolved information. Serum metabolites serve as the gold-standard noninvasive modality for NAFLD diagnosis (Kundu et al., 2023). Given that NAFLD is a prevalent metabolic disorder, we hypothesized that serum metabolomic profiles could more directly and accurately reflect disease status compared with existing methodologies. To test this hypothesis, 165 NAFLD patients and 26 healthy controls were enrolled in this study. Via non-targeted metabolomics, a total of 942 differentially expressed metabolites were identified using the criteria of VIP >1 and *P* < 0.05. Pathway analysis of these metabolites revealed perturbations in cholesterol metabolism, caffeine metabolism, and the FoxO/AMPK signaling axes in NAFLD patients, corroborating prior findings.

Cholesterol, a pivotal lipid molecule in animal cells, maintains the cellular barrier function and serves as a key metabolic precursor for bile acid, vitamin D3, and steroid hormone synthesis (Luo et al., 2020). Systemic cholesterol homeostasis is regulated via two primary pathways: exogenous dietary cholesterol intake and endogenous cholesterol biosynthesis (Brown et al., 1986). Dysregulated cholesterol metabolism is a hallmark of NAFLD pathogenesis (Arguello et al., 2015). As the central organ for lipid synthesis, gluconeogenesis, and cholesterol metabolism, the liver’s excessive cholesterol accumulation can induce hepatic steatosis. Moreover, free cholesterol accumulation resulting from impaired cholesterol transport or metabolic imbalance exacerbates NAFLD progression (Li et al., 2021). Inhibiting exogenous cholesterol absorption has been shown to reduce hepatic cholesterol levels, thereby mitigating NAFLD incidence (Romero-Gomez et al., 2017). Additionally, cholesterol overload disrupts mitochondrial and endoplasmic reticulum function, induces hepatocyte apoptosis and necrosis, and ultimately amplifies liver injury (Mendez-Sanchez et al., 2018). Caffeine metabolism is also implicated in NAFLD pathogenesis, with its metabolites emerging as potential biomarkers for disease progression (Coelho et al., 2022). Multiple investigations have demonstrated that caffeine and certain metabolites exhibit antioxidant properties, safeguarding cells against oxidative damage and conferring salutary effects (Martini et al., 2016; Rathod et al., 2013). Dual-specific phosphatase nine exerts hepatoprotection by directly inhibiting apoptosis signal-regulating kinase 1 (ASK1) and downstream kinases P38 and c-Jun N-terminal kinase (JNK) (Ye et al., 2019). An animal study revealed that caffeine treatment significantly upregulates Dusp9 expression, further suppressing ASK1-P38/JNK pathway activation and associated gene dysregulation (Xin et al., 2025). These pleiotropic effects contribute to caffeine’s therapeutic efficacy in reducing hepatic lipid deposition, reversing inflammation and fibrosis, and ameliorating metabolic-associated steatohepatitis (MASH). Caffeine has been validated as a common therapeutic target across diverse pathologies, including metabolic syndrome, diabetes, liver fibrosis, and even hepatocellular carcinoma (Setiawan et al., 2015; Molloy et al., 2012; Ding et al., 2014). FoxO transcription factors orchestrate diverse physiological and pathological processes, including cellular homeostasis, metabolism, oncogenesis, and cardiovascular regulation (Santo et al., 2023). FoxO1, a pivotal regulator of hepatic gluconeogenesis and lipid metabolism, plays a dual role in lipid and cholesterol homeostasis. While its activation mitigates liver injury and slows NAFLD progression (Tian et al., 2024), Matsumoto et al. reported that hepatic FoxO1 overexpression exacerbates steatosis by promoting triglyceride synthesis and suppressing fatty acid oxidation (Matsumoto et al., 2006). The SIRT1/FoxO1 axis is implicated in cholesterol metabolism, wherein SIRT1 deacetylates and activates FoxO1, elevating its protein levels to modulate oxidative stress and inflammatory responses (Li et al., 2017). Cholesterol efflux, the process by which free cholesterol is exported from foam cells via transporters and assimilated by high-density lipoprotein or apolipoprotein A-1, is critical for cholesterol homeostasis (Li et al., 2017). ATP-binding cassette subfamily A member 1 (ABCA1), a key mediator of cholesterol efflux, is transcriptionally regulated by FoxO1. By facilitating hepatocellular cholesterol efflux to HDL, ABCA1 maintains systemic cholesterol balance (Li et al., 2021). AMPK, a serine/threonine kinase belonging to the heterotrimeric protein kinase family, orchestrates diverse metabolic processes, including glucose, lipid, and protein metabolism (Steinberg and HARDIE, 2023). Under energy stress, AMPK activates catabolic pathways (e.g., glycolysis, tricarboxylic acid cycle, and fatty acid oxidation) to generate energy, while suppressing anabolic processes such as fatty acid, protein, and cholesterol synthesis. It also preserves energy homeostasis by inactivating mammalian target of rapamycin complex one to limit energy expenditure (Laplante and Sabatini, 2009). Experimental evidence indicates that genetic or pharmacological activation of AMPK ameliorates NAFLD (Esquejo et al., 2018; Garcia et al., 2019). Reduced AMPK expression has been observed in NAFLD mouse models and free fatty acid-induced HepG2 cells [39309511]. In liver tissues from NASH patients, Western blotting and immunohistochemistry analyses revealed significantly decreased AMPK phosphorylation, concomitant with elevated levels of the activated pro-apoptotic protein cleaved caspase-6, compared to healthy controls. Further investigations demonstrated a negative correlation between AMPK activity and caspase-6 activation, suggesting that AMPK exerts hepatoprotective anti-apoptotic effects by inhibiting caspase-6 activation (Zhao et al., 2020). Ryan et al. reported that the AMPK β1 activator PF-06409577 reduced hepatic and systemic lipid/cholesterol levels in preclinical rodent and primate models (Esquejo et al., 2018). In recent years, multiple drugs have been shown to alleviate hepatic inflammation, oxidative stress, regulate lipid metabolism, enhance autophagy, and modulate mitochondrial biogenesis via AMPK signaling pathway modulation (Cai et al., 2025). Thus, AMPK is recognized as a promising therapeutic target for metabolic diseases (Esquejo et al., 2018).

**FIGURE 7:**
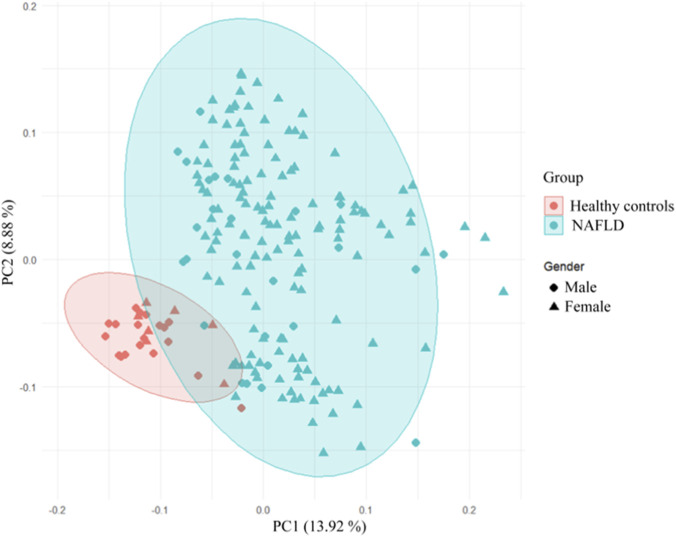
Association between grouping and gender.

The integration of metabolomics with machine learning algorithms, which excel at identifying sample patterns and consensus marker molecules from high-throughput data, has emerged as a pivotal tool for discovering metabolic biomarkers in disease diagnosis (Shen et al., 2021). Numerous studies have validated the utility of machine learning in NAFLD diagnosis: for instance, the RF model exhibits superior performance in early fatty liver disease diagnosis (Wu et al., 2019), and its combination with metabolomics effectively optimizes clinical decision-making (Ma et al., 2018; Buergel et al., 2022), supporting the methodological validity of this study. It is noteworthy that despite a significant gender imbalance between the NAFLD group and healthy controls in this study, rigorous statistical validation confirmed the validity of the current grouping design. Comparative analyses of linear models with and without gender as a covariate revealed that 83% (786/942) of the significant intergroup differences in metabolites remained consistent. This finding indicates that gender had no substantial impact on the majority of metabolite group differences. Only 16.2% (153/942) of metabolites showed false-positive results attributable to gender confounding, and such interference could be effectively mitigated by incorporating gender as a covariate in the models. Furthermore, principal component analysis (PCA) demonstrated a distinct separation trend between the two groups in the principal component space, suggesting that the intergroup differences in metabolic phenotypes themselves constitute the primary signal within the dataset (Figure 7).

Serum metabolite-based diagnosis offers advantages including independence from operator expertise, higher sensitivity, and specificity, thereby showing promising application prospects in large-scale screening, early identification, and diagnosis of NAFLD. This study mitigated the overfitting risk arising from 942 differentially expressed metabolites identified via untargeted metabolomics and a sample size of 191. By elevating the VIP threshold of the OPLS-DA model to 2.8 for dimensionality reduction, 19 core metabolites were ultimately selected. This strategy retained the classification contribution of metabolites while ensuring model robustness by reducing feature redundancy, laying a reliable foundation for subsequent machine learning analyses. Based on the selected core features, validation of six machine learning models (KNN, RF, SVM, GNB, LR, DT) revealed that the LR model exhibited flawless performance in both training and testing sets, with the remaining models also showing strong predictive capacity, confirming the reliable diagnostic value of the selected metabolites for NAFLD. Further analysis using interpretable methods demonstrated that 1-methyluric acid contributed most significantly to model decision-making and exerted marked nonlinear synergistic effects with maresin 1, canavaninosuccinate, and paraxanthine. This indicates that NAFLD-related metabolic perturbations result not from a single metabolic aberration but from a metabolic network imbalance. Box plot analyses confirmed that 1-Methyluric acid was downregulated in the NAFLD group, while the other three metabolites were upregulated (P < 0.0001). This phenotypic discrepancy is consistent with the model screening results, validating the robustness of these four metabolites as biomarkers and providing a clear metabolic phenotypic basis for distinguishing NAFLD from healthy controls. Several studies have demonstrated that caffeine metabolites can exert beneficial effects by directly regulating redox balance (Eichwald et al., 2023). As a key intermediate in caffeine metabolism, 1-methyluric acid, despite its considerable oxidative activity, can reduce lipid peroxide production by inhibiting the oxidative modification of human low-density lipoprotein, thereby indirectly alleviating liver damage (Lee, 2000). Paraxanthine is one of the primary metabolites of caffeine in the body, and its production is dependent on cytochrome P450 enzymes, particularly cytochrome P450 2E1 (CYP2E1) within this family. Research has indicated that, compared with healthy individuals, patients with NAFLD exhibit significantly upregulated hepatic CYP2E1 expression, and this enzyme promotes NAFLD progression by exacerbating oxidative stress (Abdelmegeed et al., 2012). Given that abnormal CYP2E1 activity may affect the efficiency of paraxanthine production, changes in its levels could serve as a potential indicator of liver metabolic status. maresin 1, the first identified anti-inflammatory and pro-resolving mediator in the maresin family, is primarily synthesized by M2-type macrophages; it can inhibit lipid accumulation in hepatocytes and improve insulin resistance. A cross-sectional study suggested that decreased maresin one levels may be involved in the pathogenesis of NAFLD. Notably, in the present study, serum maresin one levels were elevated in NAFLD patients. A plausible explanation for this phenomenon is that, as an anti-inflammatory mediator, maresin 1 may be compensatorily upregulated in the early stage of simple steatosis to mitigate hepatocyte damage. Furthermore, studies have confirmed that exogenous supplementation of maresin one can inhibit endoplasmic reticulum stress and ameliorate hepatic steatosis in a high-fat diet-induced NASH mouse model by activating the AMPK/SERCA2b pathway, suggesting that endogenous upregulation may represent a self-protective mechanism of the body. canavaninosuccinate is an organic acid metabolite synthesized and metabolized in mammalian liver tissue. Studies have shown that its expression is significantly higher in patients with liver cancer compared to healthy controls, while it is significantly lower in cirrhotic patients, indicating that it may be involved in metabolic disorders under pathological liver conditions (Wang et al., 2012).

However, this study still has several limitations. First, due to ethical and practical constraints, liver biops—the gold standard for NAFLD diagnosis—was not employed for patient stratification. Instead, ultrasound examination was selected as an alternative diagnostic method in accordance with clinical guidelines. Additionally, constrained by current clinical resources, this study did not utilize MRI-PDFF technology to quantitatively stratify NAFLD based on the severity of steatosis, inflammation, or fibrosis. Future research should integrate such quantitative imaging approaches to improve the precision of disease stratification. Second, the sensitivity and specificity of non-targeted metabolomics require further improvement: although validated using multiple machine learning models in this study, additional algorithm optimization is still needed to identify more precise biomarkers. Furthermore, due to the limited availability of NAFLD metabolomics datasets with consistent experimental protocols and matched population characteristics, external independent cohort validation was not conducted. This limitation prevents a comprehensive assessment of the generalizability of the proposed models and biomarkers. Future research should include larger sample sizes and multicenter data, combined with liver biopsy validation and external independent cohort validation, to comprehensively enhance the clinical translational value of the findings.

This study demonstrates that integrated serum metabolomics and machine learning analyses offer high reliability in identifying diagnostic biomarkers for NAFLD and elucidating its pathophysiological mechanisms. The four differentially expressed metabolites (maresin 1, canavaninosuccinate, paraxanthine, and 1-methyluric acid) identified and validated through multi-model screening exhibit potential as both NAFLD diagnostic biomarkers and therapeutic targets, with 1-Methyluric acid emerging as the core biomarker with the highest diagnostic efficacy. While it cannot be definitively concluded that these metabolites can replace invasive diagnostic modalities such as liver biopsy, the findings provide scientific evidence for advancing non-invasive NAFLD diagnostic technologies, as well as for early prevention, detection, and diagnosis.

## Data Availability

The datasets presented in this study can be found in online repositories. The names of the repository/repositories and accession number(s) can be found in the article/Supplementary Material.
