# Supplementary material for: Serum biomarker screening and metabolic profiling analysis of nonalcoholic fatty liver disease patients using untargeted metabolomics and machine learning techniques
Source: Front Mol Biosci. 2026 Feb 9;13:1730023. doi: 10.3389/fmolb.2026.1730023 (PMC12926098; doi:10.3389/fmolb.2026.1730023)
Supplement: Supplementary file 1 [file Supplementaryfile1.docx]

**Supplementary Files**

**Supplementary** **Table 1. Identification of serum metabolites in NAFLD patients and healthy controls**

| **No.** | **Compound name** | **Precursor type** | **Formula** | **KEGG ID** | **FC** | **VIP** | ***P* value** | **Trend in NAFLD** |
| --- | --- | --- | --- | --- | --- | --- | --- | --- |
| 1 | 2-Oxazolidinone | [M+Na]+ | C_3_H_5_NO_2_ | C06695 | 1.79 | 3.232651 | 1.14E-20 | up |
| 2 | 3-Chlorocatechol | [M-NH3+H]+ | C_6_H_5_ClO_2_ | C05618 | 1.79 | 3.198385 | 2.26E-20 | up |
| 3 | Trifluoromethanesulfonamide | [M-H2O+H]+ | CH_2_F_3_NO_2_S | - | 2.29 | 3.171178 | 4.35E-21 | up |
| 4 | 3-Chlorobenzoate | [M-H2O+H]+ | C_7_H_4_ClO_2_- | - | 2.55 | 3.378533 | 9.49E-10 | up |
| 5 | 4-Chlorobenzoic acid | [M-NH3+H]+ | C_7_H_5_ClO_2_ | C02370 | 1.71 | 2.945293 | 4.91E-16 | up |
| 6 | Pentafluorobenzene | [M+H]+ | C_6_HF_5_ | - | 1.5 | 2.882985 | 9.18E-18 | up |
| 7 | 2-(3-Methylthio)propylmalate | [M+H]+ | C_8_H_14_O_5_S | C17214 | 0.19 | 3.20947 | 2.09E-23 | down |
| 8 | Stattic | [M+Na]+ | C_8_H_5_NO_4_S | - | 4.03 | 3.069792 | 8.84E-20 | up |
| 9 | beta-D-Glucose 6-phosphate | [M-NH3+H]+ | C_6_H_13_O_9_P | C01172 | 0.7 | 2.984968 | 6.02E-07 | down |
| 10 | Canavaninosuccinate | [M-H2O+H]+ | C_9_H_16_N_4_O_7_ | - | 1.73 | 2.997263 | 7.08E-18 | up |
| 11 | 3-Ketosphingosine | [M+H]+ | C_18_H_35_NO_2_ | - | 2.28 | 2.956325 | 2.95E-19 | up |
| 12 | Paraxanthine | [2M+H]+ | C_7_H_8_N_4_O_2_ | C13747 | 3.57 | 3.172232 | 4.2E-10 | up |
| 13 | Pelargonic acid | [M-H]- | C_9_H_18_O_2_ | C01601 | 0.42 | 2.882114 | 5.52E-47 | down |
| 14 | Aesculetin | [M+HCOO]- | C_9_H_6_O_4_ | C09263 | 0.4 | 2.911791 | 4.67E-14 | down |
| 15 | 2-Chloromaleylacetate | [M+HCOO]- | C_6_H_5_C_l_O_5_ | C06329 | 0.53 | 2.965357 | 3.82E-19 | down |
| 16 | 6-Hydroxymellein | [M+HCOO]- | C_10_H_10_O_4_ | C02379 | 0.39 | 2.916456 | 2.47E-14 | down |
| 17 | 1-Methyluric acid | [M+CH3CO2]- | C_6_H_6_N_4_O_3_ | C16359 | 0.38 | 2.946835 | 4.21E-14 | down |
| 18 | Maresin 1 | [M-H]- | C_22_H_32_O_4_ | - | 4.47 | 3.366606 | 6.17E-20 | up |
| 19 | Delcorine | [M-H]- | C_26_H_41_NO_7_ | C08675 | 0.24 | 3.473073 | 6.13E-22 | down |

Abbreviations: No., Number; KEGG, Kyoto Encyclopedia of Genes and Genomes; FC, fold change; VIP, variable importance in projection; NAFLD, Non-Alcoholic Fatty Liver Disease.

**Supplementary Table 2. Metadata of serum samples from NAFLD patients and healthy controls**

| Sample ID | Group | Age/year | Sex |
| --- | --- | --- | --- |
| 01 | NAFLD | 27 | Male |
| 02 | NAFLD | 39 | Male |
| 03 | NAFLD | 55 | Female |
| 04 | NAFLD | 32 | Male |
| 05 | NAFLD | 66 | Male |
| 06 | NAFLD | 36 | Male |
| 07 | NAFLD | 51 | Male |
| 08 | NAFLD | 34 | Male |
| 09 | NAFLD | 43 | Male |
| 10 | NAFLD | 51 | Male |
| 11 | NAFLD | 38 | Male |
| 12 | NAFLD | 26 | Male |
| 13 | NAFLD | 22 | Female |
| 14 | NAFLD | 36 | Male |
| 15 | NAFLD | 45 | Male |
| 16 | NAFLD | 35 | Male |
| 17 | NAFLD | 37 | Male |
| 18 | NAFLD | 37 | Male |
| 19 | NAFLD | 38 | Male |
| 20 | NAFLD | 28 | Male |
| 21 | NAFLD | 34 | Male |
| 22 | NAFLD | 30 | Male |
| 23 | NAFLD | 31 | Male |
| 24 | NAFLD | 33 | Male |
| 25 | NAFLD | 35 | Male |
| 26 | NAFLD | 39 | Female |
| 27 | NAFLD | 29 | Male |
| 28 | NAFLD | 29 | Male |
| 29 | NAFLD | 32 | Male |
| 30 | NAFLD | 38 | Male |
| 31 | NAFLD | 44 | Male |
| 32 | NAFLD | 42 | Male |
| 33 | NAFLD | 41 | Male |
| 34 | NAFLD | 32 | Male |
| 35 | NAFLD | 30 | Male |
| 36 | NAFLD | 51 | Male |
| 37 | NAFLD | 36 | Male |
| 38 | NAFLD | 37 | Male |
| 39 | NAFLD | 35 | Male |
| 40 | NAFLD | 40 | Male |
| 41 | NAFLD | 29 | Male |
| 42 | NAFLD | 30 | Male |
| 43 | NAFLD | 46 | Male |
| 44 | NAFLD | 35 | Male |
| 45 | NAFLD | 55 | Male |
| 46 | NAFLD | 48 | Male |
| 47 | NAFLD | 32 | Male |
| 48 | NAFLD | 49 | Male |
| 49 | NAFLD | 38 | Male |
| 50 | NAFLD | 40 | Male |
| 51 | NAFLD | 25 | Male |
| 52 | NAFLD | 32 | Male |
| 53 | NAFLD | 36 | Male |
| 54 | NAFLD | 53 | Male |
| 55 | NAFLD | 37 | Male |
| 56 | NAFLD | 36 | Male |
| 57 | NAFLD | 42 | Male |
| 58 | NAFLD | 34 | Male |
| 59 | NAFLD | 42 | Male |
| 60 | NAFLD | 28 | Male |
| 61 | NAFLD | 34 | Female |
| 62 | NAFLD | 37 | Male |
| 63 | NAFLD | 60 | Female |
| 64 | NAFLD | 37 | Male |
| 65 | NAFLD | 58 | Male |
| 66 | NAFLD | 49 | Male |
| 67 | NAFLD | 36 | Female |
| 68 | NAFLD | 38 | Male |
| 69 | NAFLD | 23 | Male |
| 70 | NAFLD | 49 | Male |
| 71 | NAFLD | 38 | Male |
| 72 | NAFLD | 36 | Male |
| 73 | NAFLD | 41 | Male |
| 74 | NAFLD | 40 | Male |
| 75 | NAFLD | 50 | Male |
| 76 | NAFLD | 54 | Male |
| 77 | NAFLD | 29 | Male |
| 78 | NAFLD | 47 | Male |
| 79 | NAFLD | 34 | Female |
| 80 | NAFLD | 61 | Male |
| 81 | NAFLD | 38 | Female |
| 82 | NAFLD | 36 | Male |
| 83 | NAFLD | 50 | Male |
| 84 | NAFLD | 45 | Male |
| 85 | NAFLD | 31 | Male |
| 86 | NAFLD | 48 | Male |
| 87 | NAFLD | 34 | Male |
| 88 | NAFLD | 50 | Male |
| 89 | NAFLD | 27 | Female |
| 90 | NAFLD | 27 | Male |
| 91 | NAFLD | 35 | Male |
| 92 | NAFLD | 33 | Male |
| 93 | NAFLD | 43 | Male |
| 94 | NAFLD | 27 | Male |
| 95 | NAFLD | 58 | Male |
| 96 | NAFLD | 31 | Female |
| 97 | NAFLD | 25 | Male |
| 98 | NAFLD | 43 | Female |
| 99 | NAFLD | 39 | Female |
| 100 | NAFLD | 59 | Female |
| 101 | NAFLD | 50 | Male |
| 102 | NAFLD | 50 | Male |
| 103 | NAFLD | 33 | Female |
| 104 | NAFLD | 43 | Male |
| 105 | NAFLD | 25 | Male |
| 106 | NAFLD | 32 | Male |
| 107 | NAFLD | 41 | Male |
| 108 | NAFLD | 61 | Male |
| 109 | NAFLD | 48 | Female |
| 110 | NAFLD | 58 | Male |
| 111 | NAFLD | 53 | Male |
| 112 | NAFLD | 30 | Male |
| 113 | NAFLD | 66 | Male |
| 114 | NAFLD | 33 | Male |
| 115 | NAFLD | 41 | Male |
| 116 | NAFLD | 43 | Male |
| 117 | NAFLD | 42 | Male |
| 118 | NAFLD | 50 | Male |
| 119 | NAFLD | 36 | Female |
| 120 | NAFLD | 41 | Male |
| 121 | NAFLD | 59 | Male |
| 122 | NAFLD | 55 | Male |
| 123 | NAFLD | 44 | Male |
| 124 | NAFLD | 42 | Female |
| 125 | NAFLD | 65 | Male |
| 126 | NAFLD | 48 | Male |
| 127 | NAFLD | 42 | Male |
| 128 | NAFLD | 56 | Female |
| 129 | NAFLD | 35 | Male |
| 130 | NAFLD | 37 | Female |
| 131 | NAFLD | 38 | Female |
| 132 | NAFLD | 57 | Male |
| 133 | NAFLD | 42 | Male |
| 134 | NAFLD | 32 | Male |
| 135 | NAFLD | 36 | Male |
| 136 | NAFLD | 32 | Male |
| 137 | NAFLD | 26 | Male |
| 138 | NAFLD | 46 | Female |
| 139 | NAFLD | 42 | Male |
| 140 | NAFLD | 37 | Male |
| 141 | NAFLD | 35 | Female |
| 142 | NAFLD | 53 | Male |
| 143 | NAFLD | 39 | Male |
| 144 | NAFLD | 28 | Male |
| 145 | NAFLD | 26 | Male |
| 146 | NAFLD | 43 | Male |
| 147 | NAFLD | 42 | Male |
| 148 | NAFLD | 26 | Male |
| 149 | NAFLD | 57 | Female |
| 150 | NAFLD | 35 | Male |
| 151 | NAFLD | 50 | Male |
| 152 | NAFLD | 45 | Male |
| 153 | NAFLD | 31 | Male |
| 154 | NAFLD | 38 | Male |
| 155 | NAFLD | 35 | Female |
| 156 | NAFLD | 39 | Female |
| 157 | NAFLD | 27 | Male |
| 158 | NAFLD | 63 | Male |
| 159 | NAFLD | 37 | Male |
| 160 | NAFLD | 38 | Male |
| 161 | NAFLD | 42 | Female |
| 162 | NAFLD | 71 | Male |
| 163 | NAFLD | 48 | Male |
| 164 | NAFLD | 34 | Female |
| 165 | NAFLD | 31 | Male |
| 166 | Healthy Control | 41 | Female |
| 167 | Healthy Control | 34 | male |
| 168 | Healthy Control | 32 | Female |
| 169 | Healthy Control | 35 | Female |
| 170 | Healthy Control | 28 | male |
| 171 | Healthy Control | 27 | Female |
| 172 | Healthy Control | 36 | Female |
| 173 | Healthy Control | 38 | Female |
| 174 | Healthy Control | 41 | Female |
| 175 | Healthy Control | 31 | Female |
| 176 | Healthy Control | 27 | male |
| 177 | Healthy Control | 53 | Female |
| 178 | Healthy Control | 41 | Female |
| 179 | Healthy Control | 43 | Female |
| 180 | Healthy Control | 37 | Female |
| 181 | Healthy Control | 44 | male |
| 182 | Healthy Control | 24 | Female |
| 183 | Healthy Control | 44 | Female |
| 184 | Healthy Control | 39 | Female |
| 185 | Healthy Control | 27 | male |
| 186 | Healthy Control | 44 | Female |
| 187 | Healthy Control | 35 | Female |
| 188 | Healthy Control | 35 | male |
| 189 | Healthy Control | 28 | Female |
| 190 | Healthy Control | 42 | Female |
| 191 | Healthy Control | 43 | male |

**Supplementary Table 3: Data processing parameters for serum metabolomics analysis in NAFLD patients and healthy controls**

| Parameter | Value |
| --- | --- |
| sample_number | 191 |
| species | Serum |
| results_number | 2850 |
| normMethod | Support vector regression correction based on QC samples |
| xcms_peakpick_method | centWave |
| xcms_peakpick_ppm | 15 |
| xcms_peakpick_peakwidth | c(5, 30) |
| cms_peakpick_mzwid | 0.015 |
| xcms_peakpick_mzdiff | 0.01 |
| xcms_rtcorr_bw | 2 |

**Supplementary Table 4: Sample Injection Sequence and Quality Control Information**

| Experiment ID | Sample | Order | Batch |
| --- | --- | --- | --- |
| QC1 | QC1 | 1 | 1 |
| QC2 | QC2 | 2 | 1 |
| QC3 | QC3 | 3 | 1 |
| QC4 | QC4 | 4 | 1 |
| QC5 | QC5 | 5 | 1 |
| NP2501028070 | 166 | 6 | 1 |
| NP2501028071 | 167 | 7 | 1 |
| NP2501028072 | 168 | 8 | 1 |
| NP2501028073 | 169 | 9 | 1 |
| NP2501028074 | 170 | 10 | 1 |
| NP2501028075 | 171 | 11 | 1 |
| NP2501028076 | 172 | 12 | 1 |
| NP2501028077 | 173 | 13 | 1 |
| NP2501028078 | 174 | 14 | 1 |
| NP2501028079 | 175 | 15 | 1 |
| NP2501028080 | 176 | 16 | 1 |
| NP2501028081 | 177 | 17 | 1 |
| NP2501028082 | 178 | 18 | 1 |
| NP2501028083 | 179 | 19 | 1 |
| NP2501028084 | 180 | 20 | 1 |
| NP2501028085 | 181 | 21 | 1 |
| NP2501028086 | 182 | 22 | 1 |
| NP2501028087 | 183 | 23 | 1 |
| NP2501028088 | 184 | 24 | 1 |
| NP2501028089 | 185 | 25 | 1 |
| QC6 | QC6 | 26 | 1 |
| NP2501028090 | 186 | 27 | 1 |
| NP2501028091 | 187 | 28 | 1 |
| NP2501028092 | 188 | 29 | 1 |
| NP2501028093 | 189 | 30 | 1 |
| NP2501028094 | 190 | 31 | 1 |
| NP2501028095 | 191 | 32 | 1 |
| NP2501028096 | 01 | 33 | 1 |
| NP2501028097 | 02 | 34 | 1 |
| NP2501028098 | 03 | 35 | 1 |
| NP2501028099 | 04 | 36 | 1 |
| NP2501028100 | 05 | 37 | 1 |
| NP2501028101 | 06 | 38 | 1 |
| NP2501028102 | 07 | 39 | 1 |
| NP2501028103 | 08 | 40 | 1 |
| NP2501028104 | 09 | 41 | 1 |
| NP2501028105 | 10 | 42 | 1 |
| NP2501028106 | 11 | 43 | 1 |
| NP2501028107 | 12 | 44 | 1 |
| NP2501028108 | 13 | 45 | 1 |
| NP2501028109 | 14 | 46 | 1 |
| QC7 | QC7 | 47 | 1 |
| NP2501028110 | 15 | 48 | 1 |
| NP2501028111 | 16 | 49 | 1 |
| NP2501028112 | 17 | 50 | 1 |
| NP2501028113 | 18 | 51 | 1 |
| NP2501028114 | 19 | 52 | 1 |
| NP2501028115 | 20 | 53 | 1 |
| NP2501028116 | 21 | 54 | 1 |
| NP2501028117 | 22 | 55 | 1 |
| NP2501028118 | 23 | 56 | 1 |
| NP2501028119 | 24 | 57 | 1 |
| NP2501028120 | 25 | 58 | 1 |
| NP2501028121 | 26 | 59 | 1 |
| NP2501028122 | 27 | 60 | 1 |
| NP2501028123 | 28 | 61 | 1 |
| NP2501028124 | 29 | 62 | 1 |
| NP2501028125 | 30 | 63 | 1 |
| NP2501028126 | 31 | 64 | 1 |
| NP2501028127 | 32 | 65 | 1 |
| NP2501028128 | 33 | 66 | 1 |
| NP2501028129 | 34 | 67 | 1 |
| QC8 | QC8 | 68 | 1 |
| NP2501028130 | 35 | 69 | 1 |
| NP2501028131 | 36 | 70 | 1 |
| NP2501028132 | 37 | 71 | 1 |
| NP2501028133 | 38 | 72 | 1 |
| NP2501028134 | 39 | 73 | 1 |
| NP2501028135 | 40 | 74 | 1 |
| NP2501028136 | 41 | 75 | 1 |
| NP2501028137 | 42 | 76 | 1 |
| NP2501028138 | 43 | 77 | 1 |
| NP2501028139 | 44 | 78 | 1 |
| NP2501028140 | 45 | 79 | 1 |
| NP2501028141 | 46 | 80 | 1 |
| NP2501028142 | 47 | 81 | 1 |
| NP2501028143 | 48 | 82 | 1 |
| NP2501028144 | 49 | 83 | 1 |
| NP2501028145 | 50 | 84 | 1 |
| NP2501028146 | 51 | 85 | 1 |
| NP2501028147 | 52 | 86 | 1 |
| NP2501028148 | 53 | 87 | 1 |
| NP2501028149 | 54 | 88 | 1 |
| QC9 | QC9 | 89 | 1 |
| NP2501028150 | 55 | 90 | 1 |
| NP2501028151 | 56 | 91 | 1 |
| NP2501028152 | 57 | 92 | 1 |
| NP2501028153 | 58 | 93 | 1 |
| NP2501028154 | 59 | 94 | 1 |
| NP2501028155 | 60 | 95 | 1 |
| NP2501028156 | 61 | 96 | 1 |
| NP2501028157 | 62 | 97 | 1 |
| NP2501028158 | 63 | 98 | 1 |
| NP2501028159 | 64 | 99 | 1 |
| NP2501028160 | 65 | 100 | 1 |
| NP2501028161 | 66 | 101 | 1 |
| NP2501028162 | 67 | 102 | 1 |
| NP2501028163 | 68 | 103 | 1 |
| NP2501028164 | 69 | 104 | 1 |
| NP2501028165 | 70 | 105 | 1 |
| QC10 | QC10 | 106 | 1 |
| QC11 | QC11 | 107 | 1 |
| QC12 | QC12 | 108 | 1 |
| QC13 | QC13 | 109 | 2 |
| QC14 | QC14 | 110 | 2 |
| QC15 | QC15 | 111 | 2 |
| QC16 | QC16 | 112 | 2 |
| QC17 | QC17 | 113 | 2 |
| NP2501028167 | 71 | 114 | 2 |
| NP2501028168 | 72 | 115 | 2 |
| NP2501028169 | 73 | 116 | 2 |
| NP2501028170 | 74 | 117 | 2 |
| NP2501028171 | 75 | 118 | 2 |
| NP2501028172 | 76 | 119 | 2 |
| NP2501028173 | 77 | 120 | 2 |
| NP2501028174 | 78 | 121 | 2 |
| NP2501028175 | 79 | 122 | 2 |
| NP2501028176 | 80 | 123 | 2 |
| NP2501028177 | 81 | 124 | 2 |
| NP2501028178 | 82 | 125 | 2 |
| NP2501028179 | 83 | 126 | 2 |
| NP2501028180 | 84 | 127 | 2 |
| NP2501028181 | 85 | 128 | 2 |
| NP2501028182 | 86 | 129 | 2 |
| NP2501028183 | 87 | 130 | 2 |
| NP2501028184 | 88 | 131 | 2 |
| NP2501028185 | 89 | 132 | 2 |
| NP2501028186 | 90 | 133 | 2 |
| QC18 | QC18 | 134 | 2 |
| NP2501028187 | 91 | 135 | 2 |
| NP2501028188 | 92 | 136 | 2 |
| NP2501028189 | 93 | 137 | 2 |
| NP2501028190 | 94 | 138 | 2 |
| NP2501028191 | 95 | 139 | 2 |
| NP2501028192 | 96 | 140 | 2 |
| NP2501028193 | 97 | 141 | 2 |
| NP2501028194 | 98 | 142 | 2 |
| NP2501028195 | 99 | 143 | 2 |
| NP2501028196 | 100 | 144 | 2 |
| NP2501028197 | 101 | 145 | 2 |
| NP2501028198 | 102 | 146 | 2 |
| NP2501028199 | 103 | 147 | 2 |
| NP2501028200 | 104 | 148 | 2 |
| NP2501028201 | 105 | 149 | 2 |
| NP2501028202 | 106 | 150 | 2 |
| NP2501028203 | 107 | 151 | 2 |
| NP2501028204 | 108 | 152 | 2 |
| NP2501028205 | 109 | 153 | 2 |
| NP2501028206 | 110 | 154 | 2 |
| QC19 | QC19 | 155 | 2 |
| NP2501028207 | 111 | 156 | 2 |
| NP2501028208 | 112 | 157 | 2 |
| NP2501028209 | 113 | 158 | 2 |
| NP2501028210 | 114 | 159 | 2 |
| NP2501028211 | 115 | 160 | 2 |
| NP2501028212 | 116 | 161 | 2 |
| NP2501028213 | 117 | 162 | 2 |
| NP2501028214 | 118 | 163 | 2 |
| NP2501028215 | 119 | 164 | 2 |
| NP2501028216 | 120 | 165 | 2 |
| NP2501028217 | 121 | 166 | 2 |
| NP2501028218 | 122 | 167 | 2 |
| NP2501028219 | 123 | 168 | 2 |
| NP2501028220 | 124 | 169 | 2 |
| NP2501028221 | 125 | 170 | 2 |
| NP2501028222 | 126 | 171 | 2 |
| NP2501028223 | 127 | 172 | 2 |
| NP2501028224 | 128 | 173 | 2 |
| NP2501028225 | 129 | 174 | 2 |
| NP2501028226 | 130 | 175 | 2 |
| QC20 | QC20 | 176 | 2 |
| NP2501028227 | 131 | 177 | 2 |
| NP2501028228 | 132 | 178 | 2 |
| NP2501028229 | 133 | 179 | 2 |
| NP2501028230 | 134 | 180 | 2 |
| NP2501028231 | 135 | 181 | 2 |
| NP2501028232 | 136 | 182 | 2 |
| NP2501028233 | 137 | 183 | 2 |
| NP2501028234 | 138 | 184 | 2 |
| NP2501028235 | 139 | 185 | 2 |
| NP2501028236 | 140 | 186 | 2 |
| NP2501028237 | 141 | 187 | 2 |
| NP2501028238 | 142 | 188 | 2 |
| NP2501028239 | 143 | 189 | 2 |
| NP2501028240 | 144 | 190 | 2 |
| NP2501028241 | 145 | 191 | 2 |
| NP2501028242 | 146 | 192 | 2 |
| NP2501028243 | 147 | 193 | 2 |
| NP2501028244 | 148 | 194 | 2 |
| NP2501028245 | 149 | 195 | 2 |
| NP2501028246 | 150 | 196 | 2 |
| QC21 | QC21 | 197 | 2 |
| NP2501028247 | 151 | 198 | 2 |
| NP2501028248 | 152 | 199 | 2 |
| NP2501028249 | 153 | 200 | 2 |
| NP2501028250 | 154 | 201 | 2 |
| NP2501028251 | 155 | 202 | 2 |
| NP2501028252 | 156 | 203 | 2 |
| NP2501028253 | 157 | 204 | 2 |
| NP2501028254 | 158 | 205 | 2 |
| NP2501028255 | 159 | 206 | 2 |
| NP2501028256 | 160 | 207 | 2 |
| NP2501028257 | 161 | 208 | 2 |
| NP2501028258 | 162 | 209 | 2 |
| NP2501028259 | 163 | 210 | 2 |
| NP2501028260 | 164 | 211 | 2 |
| NP2501028261 | 165 | 212 | 2 |
| QC22 | QC22 | 213 | 2 |
| QC23 | QC23 | 214 | 2 |
| QC24 | QC24 | 215 | 2 |

**Supplementary File 1: Machine Learning Python Scripts**

import pandas as pd

from sklearn.model_selection import train_test_split, StratifiedKFold

from sklearn.neighbors import KNeighborsClassifier

from sklearn.ensemble import RandomForestClassifier

from sklearn.svm import SVC

from sklearn.naive_bayes import GaussianNB

from sklearn.linear_model import LogisticRegression

from sklearn.tree import DecisionTreeClassifier

from sklearn.metrics import (roc_curve, auc, accuracy_score, recall_score,

precision_score, f1_score)

from sklearn.preprocessing import StandardScaler

import matplotlib.pyplot as plt

import chardet

import numpy as np

import os

# The data reading and preprocessing section remains unchanged

# -------------------------- Data Reading and Preprocessing --------------------------

data_file_path = r'E:\Study\data.csv'

try:

with open(data_file_path, 'rb') as f:

raw_data = f.read(100000)

result = chardet.detect(raw_data)

detected_encoding = result['encoding']

confidence = result['confidence']

print(f"Detected data file encoding: {detected_encoding}, confidence: {confidence:.2f}")

data = pd.read_csv(data_file_path, encoding=detected_encoding)

print(f"Data loaded successfully, shape: {data.shape}")

except FileNotFoundError:

print("Error: Data file not found, please check the path")

print("\nContinuing with simulated data...")

data = pd.DataFrame(np.random.randn(191, 942), columns=[f'metabolite_{i}' for i in range(1, 943)],

index=[f'sample_{i}' for i in range(1, 192)])

except UnicodeDecodeError:

print("Error: Data file encoding is not UTF-8, trying other common encodings...")

encodings_to_try = ['gbk', 'gb2312', 'latin-1', 'utf-16']

success = False

for encoding in encodings_to_try:

try:

data = pd.read_csv(data_file_path, encoding=encoding)

print(f"Successfully read data file with {encoding} encoding")

success = True

break

except UnicodeDecodeError:

print(f"Failed to try {encoding} encoding")

if not success:

print("All encoding attempts failed, using error replacement strategy")

data = pd.read_csv(data_file_path, encoding='utf-8', errors='replace')

except Exception as e:

print(f"Error: An exception occurred while reading the data file: {e}")

print("\nContinuing with simulated data...")

data = pd.DataFrame(np.random.randn(191, 942), columns=[f'metabolite_{i}' for i in range(1, 943)],

index=[f'sample_{i}' for i in range(1, 192)])

# Read Group Table

group_file_path = r'E:\Study\group.csv'

try:

with open(group_file_path, 'rb') as f:

raw_data = f.read(100000)

result = chardet.detect(raw_data)

detected_encoding = result['encoding']

confidence = result['confidence']

print(f"Detected group file encoding: {detected_encoding}, confidence: {confidence:.2f}")

group_df = pd.read_csv(group_file_path, encoding=detected_encoding, header=None)

print(f"Group file loaded successfully, shape: {group_df.shape}")

if group_df.shape[1] < 2:

raise ValueError("Group file requires at least two columns (sample name and group)")

group_df.columns = ['sample_name', 'group']

group_counts = group_df['group'].value_counts()

print("\nGroup statistics:")

for group, count in group_counts.items():

print(f"Group {group}: {count} samples")

except FileNotFoundError:

print("Error: Group file not found, please check the path")

print("\nContinuing with default group information...")

num_samples = len(data)

group_df = pd.DataFrame({

'sample_name': [f'sample_{i}' for i in range(1, num_samples + 1)],

'group': ['A'] * 26 + ['B'] * (num_samples - 26)

})

except Exception as e:

print(f"Error: An exception occurred while reading the group file: {e}")

print("\nContinuing with default group information...")

num_samples = len(data)

group_df = pd.DataFrame({

'sample_name': [f'sample_{i}' for i in range(1, num_samples + 1)],

'group': ['A'] * 26 + ['B'] * (num_samples - 26)

})

# Data Preprocessing

print("\nData basic information:")

data.info()

print("\nDataframe columns:", data.columns.tolist())

if 'metabolite' in data.columns:

data.set_index('metabolite', inplace=True)

print(f"Set 'metabolite' column as index")

else:

first_column = data.columns[0]

data.set_index(first_column, inplace=True)

print(f"Set first column '{first_column}' as index")

data = data.T

print(f"Data transposed, new shape: {data.shape}")

# Construct Target Variable

sample_names = data.index.tolist()

y = []

matched_samples = 0

unmatched_samples = 0

unmatched_list = []

for sample in sample_names:

group_entry = group_df[group_df['sample_name'] == sample]

if not group_entry.empty:

group = group_entry.iloc[0]['group']

if group == 'A':

y.append(0) # Healthy group

elif group == 'B':

y.append(1) # Patient group

else:

print(f"Warning: Sample {sample} has unknown group {group}, defaulting to 1")

y.append(1)

matched_samples += 1

else:

print(f"Warning: Sample {sample} not found in group file, defaulting to 1")

y.append(1)

unmatched_samples += 1

unmatched_list.append(sample)

y = np.array(y)

print(f"\nMatching results:")

print(f"Successfully matched samples: {matched_samples}")

print(f"Unmatched samples: {unmatched_samples}")

if unmatched_samples > 0:

print(f"Unmatched samples: {unmatched_list}")

print(f"\nTarget variable y length: {len(y)}")

print(f"Class distribution: Healthy group (A)={np.sum(y == 0)}, Patient group (B)={np.sum(y == 1)}")

# X-y Length Matching

if len(data) != len(y):

print(f"Error: Number of rows in X ({len(data)}) does not match y ({len(y)})")

min_len = min(len(data), len(y))

data = data.iloc[:min_len]

y = y[:min_len]

print(f"Truncated data to length: {min_len}")

# Training/Test Set Splitting

X_train, X_test, y_train, y_test = train_test_split(

data, y, test_size=0.2, random_state=42, stratify=y

)

print(f"\nTraining set size: {len(X_train)}, Test set size: {len(X_test)}")

print(f"Training class distribution: Healthy={np.sum(y_train == 0)}, Patient={np.sum(y_train == 1)}")

print(f"Test class distribution: Healthy={np.sum(y_test == 0)}, Patient={np.sum(y_test == 1)}")

# -------------------------- Step 1: Feature Selection --------------------------

print("\n===== Step 1: Feature Selection with Random Forest =====")

rf_feature = RandomForestClassifier(random_state=42)

rf_feature.fit(X_train, y_train)

importances = rf_feature.feature_importances_

feature_names = data.columns

feature_importance = pd.Series(importances, index=feature_names).sort_values(ascending=False)

print('\nTop 10 features by importance:')

print(feature_importance.head(10))

# Plot a bar chart of feature importance for the top 10 metabolites

plt.figure(figsize=(12, 8))

top10_features = feature_importance.head(10)

bars = plt.bar(top10_features.index, top10_features.values, color='skyblue')

# Add value labels

for bar in bars:

height = bar.get_height()

plt.text(bar.get_x() + bar.get_width()/2., height + 0.001,

f'{height:.4f}', ha='center', va='bottom', rotation=45)

plt.title('Top 10 Metabolite Features by Importance', fontsize=14)

plt.xlabel('Metabolite', fontsize=12)

plt.ylabel('Importance Score', fontsize=12)

plt.xticks(rotation=45, ha='right') # Rotate labels to prevent overlap

plt.tight_layout() # Adjust layout

plt.grid(axis='y', alpha=0.3)

plt.savefig('top10_metabolite_importance.png', dpi=300)

plt.show()

top_n = 10

top_features = feature_importance.head(top_n).index.tolist()

print(f"\nSelected top {top_n} features: {top_features}")

X_train_selected = X_train[top_features]

X_test_selected = X_test[top_features]

print(f"Selected features shape - Train: {X_train_selected.shape}, Test: {X_test_selected.shape}")

# Standardization

scaler = StandardScaler()

X_train_scaled = scaler.fit_transform(X_train_selected)

X_test_scaled = scaler.transform(X_test_selected)

# -------------------------- Model Training and Metric Calculation --------------------------

print("\n===== Step 2: Model Training and Metric Calculation =====")

models = {

'KNN': KNeighborsClassifier(),

'RF': RandomForestClassifier(random_state=42),

'SVM': SVC(random_state=42, probability=True),

'GNB': GaussianNB(),

'LR': LogisticRegression(random_state=42, max_iter=5000),

'DT': DecisionTreeClassifier(random_state=42)

}

# Store metrics and ROC data (training set + test set)

metrics = {

'train': { # Training set metrics

'Accuracy': {}, 'Sensitivity': {}, 'PPV': {}, 'NPV': {}, 'F1': {}, 'AUC': {}

},

'test': { # Test set metrics

'Accuracy': {}, 'Sensitivity': {}, 'Specificity': {}, 'PPV': {}, 'NPV': {}, 'F1': {}, 'AUC': {}

}

}

roc_data = {

'train': {'fpr': {}, 'tpr': {}}, # Training set ROC data

'test': {'fpr': {}, 'tpr': {}} # Test set ROC data

}

cv_results = {model: {'accuracy': [], 'sensitivity': [], 'specificity': [], 'roc_auc': []} for model in models}

# Calculate metrics and ROC curves for the training set and test set

for model_name in models:

model = models[model_name]

try:

# Select data (standardized/original)

if model_name in ['LR', 'SVM', 'KNN']:

X_train_use = X_train_scaled

X_test_use = X_test_scaled

else:

X_train_use = X_train_selected

X_test_use = X_test_selected

# Train the model

model.fit(X_train_use, y_train)

# -------------------------- Training set evaluation --------------------------

y_pred_train = model.predict(X_train_use)

# Calculate probabilities for ROC curve generation

if hasattr(model, 'predict_proba'):

y_prob_train = model.predict_proba(X_train_use)[:, 1]

else:

y_prob_train = model.decision_function(X_train_use)

# Calculate the ROC for the training set

fpr_train, tpr_train, _ = roc_curve(y_train, y_prob_train)

roc_auc_train = auc(fpr_train, tpr_train)

# Store the training set metrics and ROC data

metrics['train']['Accuracy'][model_name] = accuracy_score(y_train, y_pred_train)

metrics['train']['Sensitivity'][model_name] = recall_score(y_train, y_pred_train)

metrics['train']['PPV'][model_name] = precision_score(y_train, y_pred_train)

metrics['train']['NPV'][model_name] = precision_score(y_train, y_pred_train, pos_label=0)

metrics['train']['F1'][model_name] = f1_score(y_train, y_pred_train)

metrics['train']['AUC'][model_name] = roc_auc_train

roc_data['train']['fpr'][model_name] = fpr_train

roc_data['train']['tpr'][model_name] = tpr_train

# -------------------------- Test set evaluation --------------------------

y_pred_test = model.predict(X_test_use)

if hasattr(model, 'predict_proba'):

y_prob_test = model.predict_proba(X_test_use)[:, 1]

else:

y_prob_test = model.decision_function(X_test_use)

# Calculate the ROC for the test set

fpr_test, tpr_test, _ = roc_curve(y_test, y_prob_test)

roc_auc_test = auc(fpr_test, tpr_test)

# Store the test set metrics and ROC data

metrics['test']['Accuracy'][model_name] = accuracy_score(y_test, y_pred_test)

metrics['test']['Sensitivity'][model_name] = recall_score(y_test, y_pred_test)

metrics['test']['Specificity'][model_name] = recall_score(y_test, y_pred_test, pos_label=0)

metrics['test']['PPV'][model_name] = precision_score(y_test, y_pred_test)

metrics['test']['NPV'][model_name] = precision_score(y_test, y_pred_test, pos_label=0)

metrics['test']['F1'][model_name] = f1_score(y_test, y_pred_test)

metrics['test']['AUC'][model_name] = roc_auc_test

roc_data['test']['fpr'][model_name] = fpr_test

roc_data['test']['tpr'][model_name] = tpr_test

print(f"{model_name} - Training and test metrics with ROC calculated")

except Exception as e:

print(f"{model_name} error: {e}")

# Cross-validation (for box plot visualization)

kfold = StratifiedKFold(n_splits=10, shuffle=True, random_state=42)

for model_name in models:

model = models[model_name]

try:

X_train_use = X_train_scaled if model_name in ['LR', 'SVM', 'KNN'] else X_train_selected.values

for train_idx, val_idx in kfold.split(X_train_use, y_train):

X_tr, X_val = X_train_use[train_idx], X_train_use[val_idx]

y_tr, y_val = y_train[train_idx], y_train[val_idx]

model.fit(X_tr, y_tr)

y_pred = model.predict(X_val)

y_prob = model.predict_proba(X_val)[:, 1] if hasattr(model, 'predict_proba') else model.decision_function(

X_val)

fpr, tpr, _ = roc_curve(y_val, y_prob)

cv_results[model_name]['accuracy'].append(accuracy_score(y_val, y_pred))

cv_results[model_name]['sensitivity'].append(recall_score(y_val, y_pred))

cv_results[model_name]['specificity'].append(recall_score(y_val, y_pred, pos_label=0))

cv_results[model_name]['roc_auc'].append(auc(fpr, tpr))

print(f"{model_name} cross-validation completed")

except Exception as e:

print(f"{model_name} CV error: {e}")

# -------------------------- Visualization 1: Line plot of training set metrics --------------------------

print("\n===== Plot 1: Training Set Metrics (Line Plot) =====")

plt.figure(figsize=(10, 6))

colors = ['blue', 'green', 'red', 'purple', 'orange', 'brown']

markers = ['o', 's', '^', 'D', 'v', '<']

for i, metric in enumerate(metrics['train']):

values = [metrics['train'][metric][model] for model in models]

plt.plot(

list(models.keys()), values,

label=metric,

marker=markers[i],

color=colors[i],

alpha=0.7,

linewidth=2

)

plt.title('Training Set Performance Metrics')

plt.xlabel('Models')

plt.ylabel('Score (0-1)')

plt.ylim(0, 1.05)

plt.legend(loc='best')

plt.grid(alpha=0.3)

plt.tight_layout()

plt.savefig('training_set_metrics_lineplot.png')

plt.show()

# -------------------------- Visualization 2: Line plot of test set metrics --------------------------

print("\n===== Plot 2: Test Set Metrics (Line Plot) =====")

plt.figure(figsize=(10, 6))

# Prepare 7 colors for the 7 indicators

colors = ['blue', 'green', 'red', 'purple', 'orange', 'brown', 'gray']

# Prepare 7 markers for the 7 indicators

markers = ['o', 's', '^', 'D', 'v', '<', '>']

for i, metric in enumerate(metrics['test']):

values = [metrics['test'][metric][model] for model in models]

plt.plot(

list(models.keys()), values,

label=metric,

marker=markers[i],

color=colors[i], # Use the indices of the color list directly, matching the number of indicators

alpha=0.7,

linewidth=2

)

plt.title('Test Set Performance Metrics')

plt.xlabel('Models')

plt.ylabel('Score (0-1)')

plt.ylim(0, 1.05)

plt.legend(loc='best')

plt.grid(alpha=0.3)

plt.tight_layout()

plt.savefig('test_set_metrics_lineplot.png')

plt.show()

# -------------------------- Visualization 3: ROC curve of the training set --------------------------

print("\n===== Plot 3: Training Set ROC Curves =====")

plt.figure(figsize=(10, 8))

colors = {'KNN': 'blue', 'RF': 'green', 'SVM': 'red', 'GNB': 'purple', 'LR': 'orange', 'DT': 'brown'}

for model_name in models:

if model_name in roc_data['train']['fpr']:

fpr = roc_data['train']['fpr'][model_name]

tpr = roc_data['train']['tpr'][model_name]

auc_score = metrics['train']['AUC'][model_name]

plt.plot(fpr, tpr, color=colors[model_name], lw=2,

label=f'{model_name} (AUC = {auc_score:.3f})')

# Draw the diagonal line

plt.plot([0, 1], [0, 1], 'k--', lw=2)

plt.xlim([0.0, 1.0])

plt.xticks(np.arange(0.0, 1.1, 0.1))

plt.ylim([0.0, 1.05])

plt.yticks(np.arange(0.0, 1.1, 0.1))

plt.xlabel('False Positive Rate')

plt.ylabel('True Positive Rate')

plt.title('ROC Curves - Training Set')

plt.legend(loc="lower right")

plt.grid(alpha=0.3)

plt.tight_layout()

plt.savefig('training_set_roc.png')

plt.show()

# -------------------------- Visualization 4: ROC curve of the test set --------------------------

print("\n===== Plot 4: Test Set ROC Curves =====")

plt.figure(figsize=(10, 8))

colors = {'KNN': 'blue', 'RF': 'green', 'SVM': 'red', 'GNB': 'purple', 'LR': 'orange', 'DT': 'brown'}

for model_name in models:

if model_name in roc_data['test']['fpr']:

fpr = roc_data['test']['fpr'][model_name]

tpr = roc_data['test']['tpr'][model_name]

auc_score = metrics['test']['AUC'][model_name]

plt.plot(fpr, tpr, color=colors[model_name], lw=2,

label=f'{model_name} (AUC = {auc_score:.3f})')

# Draw the diagonal line

plt.plot([0, 1], [0, 1], 'k--', lw=2)

plt.xlim([0.0, 1.0])

plt.xticks(np.arange(0.0, 1.1, 0.1))

plt.ylim([0.0, 1.05])

plt.yticks(np.arange(0.0, 1.1, 0.1))

plt.xlabel('False Positive Rate')

plt.ylabel('True Positive Rate')

plt.title('ROC Curves - Test Set')

plt.legend(loc="lower right")

plt.grid(alpha=0.3)

plt.tight_layout()

plt.savefig('test_set_roc.png')

plt.show()

# -------------------------- Visualization 5: Cross-validation box plot --------------------------

print("\n===== Plot 5: Cross-Validation Boxplots =====")

cv_metrics = ['accuracy', 'sensitivity', 'specificity', 'roc_auc']

for metric in cv_metrics:

plt.figure(figsize=(10, 6))

data = [cv_results[model][metric] for model in models]

plt.boxplot(data, labels=models.keys(), patch_artist=True)

plt.title(f'Cross-Validation - {metric.capitalize()}')

plt.ylabel(metric.capitalize())

plt.ylim(0, 1.05)

plt.grid(axis='y', alpha=0.3)

plt.tight_layout()

plt.savefig(f'cv_{metric}_boxplot.png')

plt.show()

# -------------------------- Output the values of the indicators --------------------------

print("\n===== Training Set Metrics =====")

train_df = pd.DataFrame(metrics['train']).T

print(train_df.round(4))

print("\n===== Test Set Metrics =====")

test_df = pd.DataFrame(metrics['test']).T

print(test_df.round(4))

print("\n===== Cross-Validation Mean ± Std =====")

cv_summary = {}

for model in models:

cv_summary[model] = {

metric: f"{np.mean(cv_results[model][metric]):.4f} ± {np.std(cv_results[model][metric]):.4f}"

for metric in cv_metrics

}

print(pd.DataFrame(cv_summary).T)

print("\nAll analyses completed.")

**
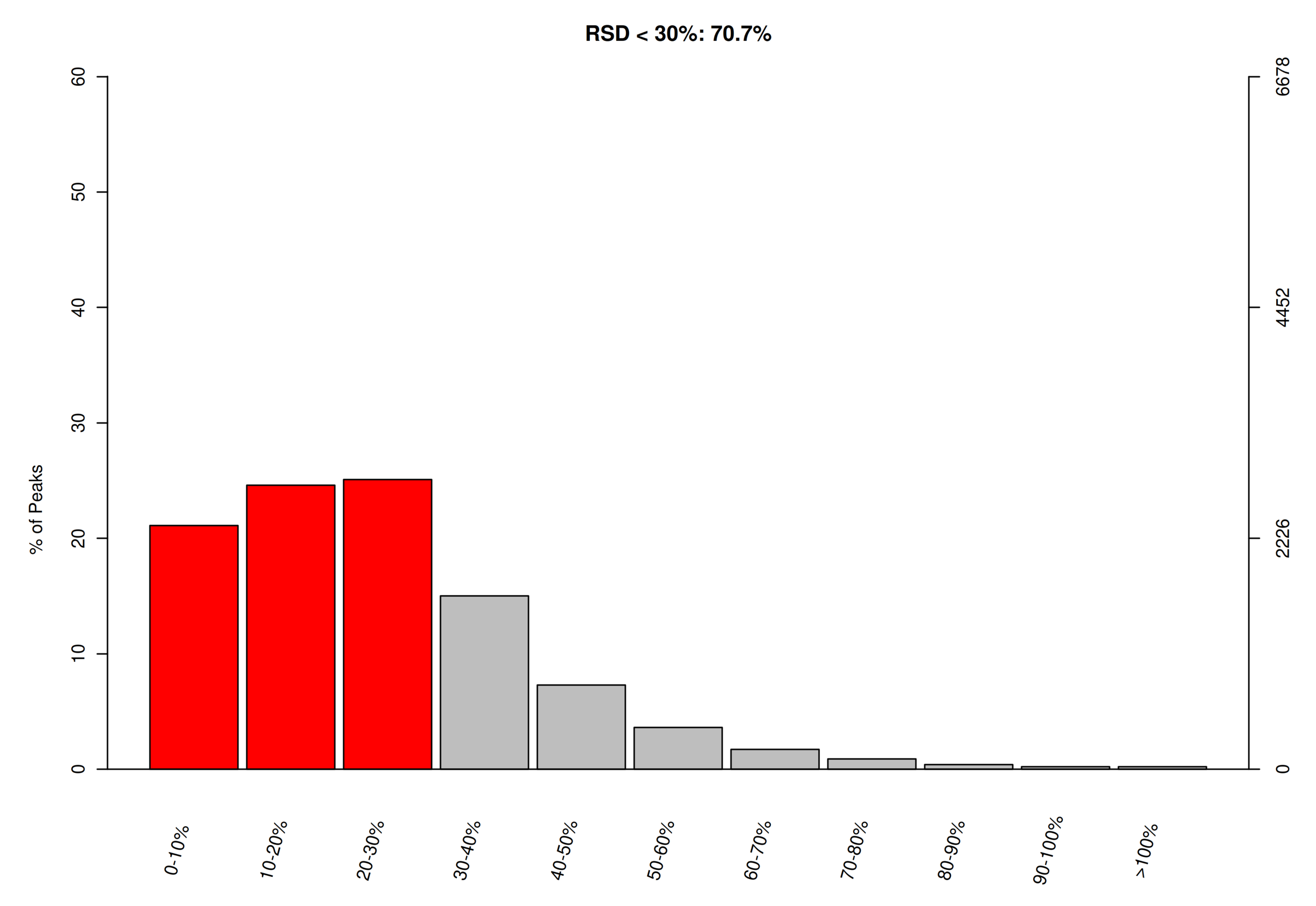
**

**Supplementary Figure 1** Metabolomics QC Sample Peak Area Relative Standard Deviation (RSD) Distribution Histogram


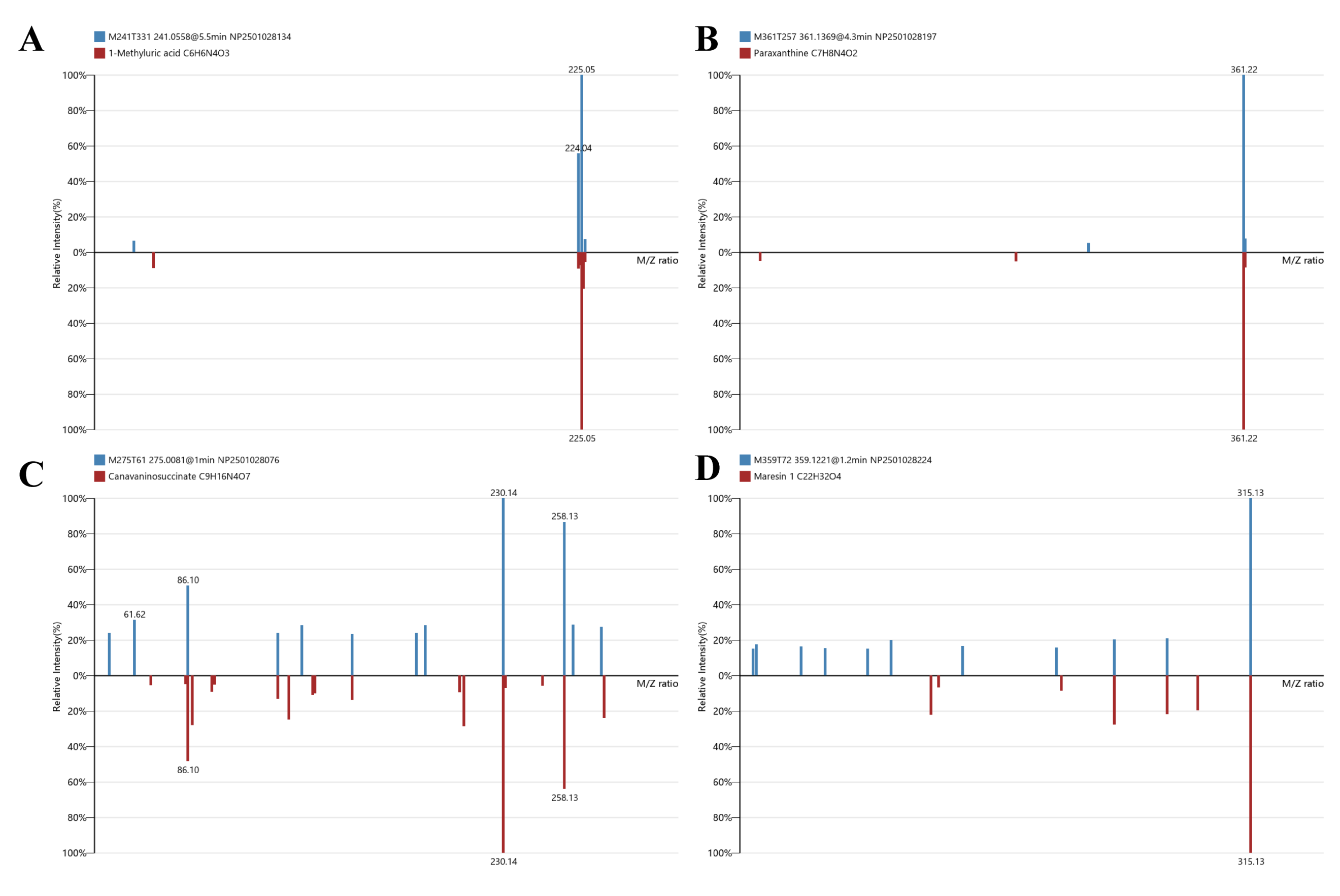


**Supplementary Figure 2** Representative MS² Spectra of Four Key Differential Metabolites Identified by Machine Learning. (A) 1-methyluric acid; (B) paraxanthine; (C) canavaninosuccinate; (D) maresin 1
